# Advances in Molecular Plant Sciences

**DOI:** 10.3390/ijms25126408

**Published:** 2024-06-10

**Authors:** Mingjian Zhou, Yanjie Xie

**Affiliations:** 1 College of Life Sciences, Nanjing Forestry University, Nanjing 210037, China; 2 College of Life Sciences, Nanjing Agricultural University, Nanjing 210095, China; 3 Department of Plant Biotechnology and Bioinformatics, Ghent University, Technologiepark 71, 9052 Ghent, Belgium; 4 VIB Center for Plant Systems Biology, 9052 Ghent, Belgium

In recent years, as biotechnological advancements have continued to unfold, our understanding of plant molecular biology has undergone a remarkable transformation. Researchers are actively exploring novel approaches to modulate both the genetic and epigenetic features of organisms, addressing issues related to plant growth, development, and environmental adaptation. With the aim of stimulating broad interest in plant molecular biology, this Special Issue of *IJMS* presents an overview of the latest fundamental discoveries in the field. These discoveries served as the foundation for the Special Issue “Advances in Molecular Plant Sciences”, which aims to provide the most current findings on novel advancements in higher plants. This Special Issue contains seven reviews, three communication articles, and thirty-six original research articles, all focusing primarily on the application of new biotechnology and elucidating different molecular mechanisms of plants throughout their life cycle and acclimatization to biotic or abiotic stress. The significant participation of numerous authors and the abundance of contributions testify to the considerable interest that the topic is currently generating within the plant science community. Here, we briefly summarize the contributions included in this Special Issue.

The life cycle of a plant is composed of a sequence of intricate biological events, such as germination, development, flowering, pollination, fertilization, and leaf senescence. All these events occur in the proper sequence and are critically regulated, ranging from gene expression to the coordination of age and environmental cues, ensuring the continuity of life and the propagation of species [1]. Xu et al. delved into pollen development, revealing how the heterologous expression of CCCH zinc-finger proteins in *Brassica campestris* can induce cytoplasmic foci formation and pollen abortion, expanding our understanding of reproductive success dynamics [2]. Zhang et al. elucidated the molecular basis of dwarfism in cotton plants by investigating the interaction between Gossypium hirsutum Tubulin beta-1 (GhTUBB1) and C-repeat-binding factor 1 (AmCBF1), offering insights into novel pathways for breeding compact cultivars [3]. Gao et al. discovered that glutathione positively contributes to the proliferation of *Pinus koraiensis* embryogenic cells [4]. In other transcriptome research, Han et al. proposed that the *SEMI-ROLLED LEAF 1 (SRL1)* gene plays a pivotal role in rice leaf development and rolling, unraveling the intricate regulatory network linked to leaf morphology and thus providing valuable perspectives for enhancing crop traits [5]. Leaf senescence plays a significant role in the growth and development of plants, which is also crucial for the survival, adaptability, and reproductive success of plants [6,7]. Through investigating the *Leaf senescence 1 (LS1)* gene in rice, Zhang et al. uncovered the role of C2H2 zinc-finger protein in regulating leaf senescence through the modulation of reactive oxygen species (ROS) production, shedding light on the molecular mechanisms underlying premature senescence and its impact on plant health [8]. In parallel, the research of Zhen et al. on foxtail millet identified key senescence-associated genes and regulatory mechanisms through a dark-induced senescence system, revealing significant physiological and transcriptomic differences between varieties with delayed and accelerated senescence, laying the groundwork for future research on leaf senescence regulation and potential genetic improvements [9]. In Arabidopsis, Wang et al. utilized the CRISPR/dCas9-TET1cd system that targeted epigenetic editing, altering the methylation status of a spontaneously occurring epiallele, demonstrating that reducing methylation levels in specific gene promoter regions can enhance gene expression, thereby influencing leaf senescence in plants [10]. Furthermore, Li et al. investigated the role of *Larix kaempferi DEFICIENS-AGAMOUS-LIKE 1 (LaDAL1)* in the life cycle of *Larix kaempferi*, uncovering its function as a time recorder and event coordinator in perennial woody plants, giving us novel insights into age recording and life cycle progression [11]. Seed size regulation, a determinant of crop yield, was also comprehensively reviewed by Zhang et al., providing valuable insights into regulatory pathways governing seed size and enhancing agricultural productivity [12]. More importantly, Wu et al. offered a thorough review of recent advancements in understanding rice seed shattering, providing a holistic perspective on this agriculturally significant trait [13]. The abovementioned studies deepen our understanding of plant life cycles from molecular mechanisms underlying key developmental processes to environmental cues shaping growth and senescence.

The synthesis of compounds within plant organisms significantly influences their growth, development, metabolic nutrition, and environmental adaptability, constituting a pivotal process for sustaining vital life activities and responding to external environmental changes [14]. The synthesis of various compounds across different plant species is tightly and meticulously regulated [15]. In fruits such as pineapple and blackberry, the identification of key genes *AcSWEET11 (Sugars Will Eventually be Exported Transporter 11)* and *RuFLS2 (Flavonol Synthase 2)* offer insights into sugar and flavonoid biosynthesis, respectively, enhancing fruit quality and nutritional value [16,17]. In peaches and barley, genes such as *PpABCC1 (ATP binding cassette 1)* and *HvWIN1 (WAX INDUCER1)* regulate anthocyanin and β-diketone biosynthesis individually, influencing fruit pigmentation and cuticle properties [18,19]. Furthermore, the elucidation of the JAZ (Jasmonate ZIM-Domain) family and CwMYC2-like protein’s roles in β-elemene biosynthesis in *Curcuma wenyujin* uncovered novel signaling pathways governing secondary metabolite production [20]. In *Arabidopsis thaliana*, chemical regulators like brassinolide and pyraclostrobin coordinate nitrogen and carbon metabolism, improving nutrient assimilation and crop productivity sustainably [21].

Biotic stresses present significant challenges to agricultural productivity worldwide [22]. Understanding the molecular mechanisms underlying plant defense responses is crucial for developing strategies to mitigate the impact of pathogens. In the study by Liang et al., the molecular basis of the hypersensitive response (HR) induced by oat dwarf virus (ODV) in tobacco plants was elucidated [23]. They identified a RING-finger protein, NbRFP1, which interacts with the ODV RepA protein, contributing to the regulation of the HR. In the investigation by researchers studying gray mold (*Botrytis elliptica*) resistance in lilies, the role of pathogenesis-related (PR) proteins was explored [24]. By analyzing the full-length transcriptome of resistant lily varieties, they identified PR proteins, particularly LhSorPR4-2, which confer resistance to *B. elliptica* infection. This study expands our understanding of plant–pathogen interactions and offers potential targets for breeding resistant lily cultivars. Additionally, the study on *Dionaea muscipula Ellis* (Venus flytrap) trichomes provided insights into the structural and functional aspects of carnivorous plant defense mechanisms [25]. By characterizing the stellate outer trichomes, the researchers suggested their role in solute transport and defense against potential pathogens. Taking advantage of integrated transcriptomic and metabolomic analyses, Sun et al. identified key pathways involved in maize resistance to fungal infection, highlighting the importance of phenylalanine metabolism in maize stalk rot caused by *Fusarium proliferatum* [26]. Xiang et al. provided a comprehensive analysis of the defense strategies rice employs against the herbivorous insect *Chilo suppressalis*, which offered valuable insights into enhancing rice’s immune response to striped stem borer, potentially reducing the reliance on chemical insecticides [27]. Collectively, these studies contribute to our understanding of plant defense mechanisms against biotic stress and offer valuable insights for developing strategies to enhance crop resilience to pathogens, facilitating the development of sustainable agricultural practices and mitigating the impact of biotic stressors on crop production.

Plants also encounter a variety of abiotic stresses, including chilling, drought, and saline–alkali conditions, throughout their survival process [28,29]. Recent research provided valuable insights into the complex mechanisms governing how plants react to abiotic stressors, offering valuable guidance for developing strategies to improve stress tolerance and enhance crop resilience. The study of Jin et al. comprehensively investigated the DNA-binding with one finger (Dof) transcription factor gene family in potato, revealing distinct stress-responsive factors and their regulatory roles under drought conditions [30]. Through genomic and transcriptomic analyses, five phylogenetic groups of Dof proteins were identified, shedding light on their potential contributions to potato development and drought stress adaptation. Through up-regulating ROS-scavenging and cold stress-related genes, the VvDREB2A transcription factor-enhanced cold tolerance in grapevines was investigated [31]. Similarly, hydrogen-rich water (HRW) mitigates chilling-induced damage in cucumber plants by boosting antioxidant enzyme activities and mitigating membrane lipid damage [32]. Additionally, Zong et al. also highlighted the importance of genetic factors in conferring alkali tolerance in soybean plants [33]. By elucidating the gene–allele system associated with alkali tolerance, this study provided a foundation for breeding soybean varieties with enhanced resilience to saline–alkali stress. Together with the comprehensive review of Cao et al., in which recent advances in understanding plant responses to saline–alkali stress were summarized, the literature further provided a roadmap for developing strategies to enhance plant resilience to saline–alkali stress [34]. Interestingly, the study by Qaseem et al. elucidated the response of *Neolamarckia cadamba* to gravistimulation, revealing the absence of a conspicuous gelatinous layer (G layer) in tension wood formation, which underscores the need for further investigation into the intricate regulatory mechanisms governing G layer synthesis and its role in shaping plant architecture and resisting gravity-induced stress [35]. Moreover, the application of plasma treatment to maize seeds emerges as a potential sustainable strategy for enhancing crop yield under field conditions, as evidenced by its positive effects on seedling emergence and plant yield [36].

Transcription factors play a central role in orchestrating complex networks underlying cellular functions and organismal development, serving as molecular switches that activate or inhibit gene expression in response to diverse signals or environmental conditions [37,38]. The comprehensive analysis of MADS-box genes across the genome of *C. chekiangoleosa* unveiled fascinating insights into the structural specificity and expression patterns of these transcription factors and shed light on their evolutionary history and functional diversity, offering valuable clues for future research on the regulation of reproductive organ development in this species [39]. Feng et al. elucidated the involvement of MYC transcription factors in the process of cucumber glandular trichome development [40]. By employing virus-induced gene silencing techniques, specific MYC transcription factors were identified as key regulators of glandular trichome development, providing novel insights into the molecular mechanisms governing this specialized plant structure. *Polygonatum cyrtonema* is a traditional Chinese medicinal herb. The discovery of WRKY transcription factors as positive regulators of polysaccharide biosynthesis unveiled the intricate regulatory mechanisms governing bioactive compound accumulation in this herb [41]. Yang et al. illustrated the diverse roles of the GRAS transcription factor family in *P. massoniana*, providing avenues for further exploration of stress response mechanisms in woody plants [42].

Proteins’ properties such as structure, stability, localization, and activity, can be modified through post-translational modifications (PTMs), allowing cells to rapidly adapt to changing conditions and finely tune cellular diverse biological processes [43,44]. Sucrose Nonfermenting 1-Related Protein Kinase 2.6 (SnRK2.6) is the core signaling component of the abscisic acid (ABA) pathway [45,46]. Through molecular dynamics simulations, Li et al. uncovered the role of PTMs such as persulfidation and phosphorylation, which finely tune the “on” and “off” states of SnRK2.6, regulating the transmission of ABA signals with precision [47]. Tang et al. identified key ubiquitination sites involved in the proteasomal degradation pathway of AtACS7 (1-aminocyclopropane-1-carboxylic acid synthase 7), a crucial enzyme in ethylene biosynthesis in Arabidopsis. Through liquid chromatography–mass spectrometry/mass spectrometry (LC-MS/MS) analysis, researchers pinpointed specific lysine residues crucial for AtACS7 ubiquitination, thereby influencing protein stability [48]. This discovery expands our understanding of post-translational regulation in ethylene signaling and offers valuable clues for dissecting the ethylene signaling pathway in plants.

Integrative analyses, such as transcriptomics, proteomics, and metabolomics, have provided unprecedented insights into the molecular mechanisms governing key agronomic traits and have revolutionized our understanding of plant biology and agricultural improvement strategies. In Masson pine, comprehensive physiological, proteomic, and gene expression analyses have unveiled the complex regulatory networks orchestrating resin yield, as well as the involvement of pathways related to carbohydrate metabolism, terpenoid biosynthesis, and hormone signaling in resin yield regulation, highlighting potential targets for enhancing resin production through molecular breeding strategies [49,50]. In cucumber grafting studies, metabolome and transcriptome analyses elucidated the molecular basis of wax loss regulation, shedding light on the intricate mechanisms underlying fruit quality modulation [51]. In rice, the identification of the *NAL22 (Narrow Leaf 22)* gene through genome-wide association studies and CRISPR/Cas9 gene editing technology unveiled novel loci for leaf width variation, paving the way for precision breeding of modern rice cultivars [52]. In chili peppers, the utilization of geminivirus-mediated gene expression manipulation to enhance capsanthin production underscores the potential of biotechnological approaches in rapidly elevating metabolite levels with enhanced nutritional and commercial value [53]. By overcoming technical challenges and leveraging bioinformatic tools, Lu et al. explored the transformative potential of single-cell chromatin accessibility profiling in plant biology, underscoring the promise of scATAC-seq in unraveling cell-type-specific gene regulatory networks [54]. In the review of Chen et al., they provided a comprehensive overview of protoplast-based approaches in plant biotechnology [55]. From protein localization studies to gene editing techniques, this review highlighted the versatility and utility of protoplast transient expression systems across diverse plant species, serving as a valuable resource for researchers in the field. In addition, the establishment of a comprehensive plant metabolite spectral library addressed a critical gap in metabolomic research. This resource facilitated metabolite identification and enables the development of pseudo-targeted methods, enhancing the efficiency and accuracy of metabolomic studies in plants [56]. The utilization of carbon quantum dots (CQDs) as biomaterials for intracellular delivery in *Arabidopsis thaliana* sheds light on their interactions with plant cells [57]. The study of Lin et al. uncovered intriguing insights into CQD transportation, epigenetic inheritance, and microRNA-mediated gene expression regulation, paving the way for future applications in plant biotechnology. Taken together, these studies showcase the power of new approaches in elucidating the genetic basis of complex traits and accelerating crop improvement efforts for enhanced resilience and productivity in agriculture.

The intricate molecular machinery orchestrating various aspects of plant biology continues to captivate researchers, driving exploration into the fundamental mechanisms underlying essential processes. In a study on citrus vein enation virus (CVEV), a new isolate, CVEV-DT1, was identified and characterized, shedding light on its genomic variation and the functional properties of its encoded proteins [58]. Combing high-throughput and traditional sequencing techniques, Dou et al. provided insights into the evolutionary relationships among CVEV isolates and highlighted the roles of specific viral proteins in host–pathogen interactions. In cotton, comparative physiological and transcriptomic analyses unraveled the distinct responses of leaves to two commonly used chemical defoliants, thidiazuron and ethephon. This research delineated the hormonal and transcriptional changes induced by thidiazuron and leaf abscission, providing valuable insights for improving defoliation strategies in cotton cultivation [59]. A comprehensive review delved into the molecular machinery of lipid droplet degradation and turnover in plants [60]. This review highlighted recent advances in understanding the pathways involved in lipid droplet degradation, with a focus on lipolysis and lipophagy, and outlined the potential implications for seed germination and plant growth.

Collectively, this Special Issue highlights the significant advancements in plant molecular biology and biotechnology, showcasing the diverse array of studies elucidating fundamental mechanisms governing plant growth, development, stress responses, and defense mechanisms. These studies not only deepen our understanding of plant biology but also offer promising avenues for enhancing crop resilience, productivity, and nutritional value through innovative biotechnological approaches. The breadth and depth of the research presented in this Special Issue underscores the importance of continued exploration in plant science to address global challenges in agriculture and food security.
